# Specific sublingual immunotherapy with peach LTP (Pru p 3). One year treatment: a case report

**DOI:** 10.1186/1757-1626-2-6553

**Published:** 2009-05-12

**Authors:** Celso Pereira, Borja Bartolomé, Juan Andrés Asturias, Iñaki Ibarrola, Beatriz Tavares, Graça Loureiro, Daniel Machado, Celso Chieira

**Affiliations:** 1 Immunoallergology Department, Coimbra University HospitalApartado 9057, 3001-301 CoimbraPortugal; 2 I & D Department, Bial-AristeguiAlameda de Urquijo 27, 48008 BilbaoSpain

## Abstract

**Introduction:**

Food allergy is an increasing problem with limited therapeutic approaches apart from to the eviction diet.

**Case presentation:**

A 40-year-old female patient with food allergy symptoms was polysensitized to almost all vegetable food since the age of 36; the onset of symptoms was during pregnancy. The allergological study demonstrated positive skin prick tests (SPT) to nuts, legumes, cereals, spices, several fresh fruits including peach, and other groups of vegetable foods however, it was negative to common aeroallergens. Serum specific IgE levels were negative (<0.35 kU/L) to profilin and carbohydrate determinants, but positive to Pru p 3 (3.5 kU/L). Positive double-blind placebo-controlled food challenge to peach confirmed the allergic disease. She received specific sublingual immunotherapy with native Pru p 3 at a concentration of 40 μg/ml with 5 administrations per week and a cumulative dose of 200 μg of nPru p 3 per month. After an ultra-rush build-up phase concluded in one day she continued therapy during a year with 5 administrations per week. The clinical evolution and laboratory studies demonstrated an early reduction on SPT reactions with no relevant changes on serum specific IgE, IgG, IgG_1_ and IgG_4_ to Pru p 3 during the immunotherapy period. The challenge test was negative 4 months after the beginning of the SLIT. Regarding clinical response she markedly improved after the first month of treatment, and by the 3th month she had no major vegetable dietary restrictions, except for nuts and pepper.

**Conclusion:**

These results demonstrate the excellent efficacy and safety of sublingual specific protein immunotherapy developed according to the patient specific sensitivity profile to Pru p3.

## Introduction

Food allergy is an increasing problem in the western countries, confirmed by several studies in children and adult patients [1]. In adult population the prevalence of food allergy varies among geographical areas, possibly in relation to local dietary habits and pollen exposures [2]. In Mediterranean region and Southern Europe fruits from Rosaceae family, including apple, peach and pear are frequently linked to clinical allergic reactions [3]. In addition to the specific allergens identified from each food other proteins are able to induce sensitization and specific IgE response [2].

Lipid transfer proteins (LTP) form a broad and widespread group of proteins, particularly of plant origin [4]. They represent a class of ubiquitous allergens specific for foods such as fresh fruits and cereals, and responsible for allergy reactions favours because of its stable and resistant structure to harsh physicochemical conditions with several well-conserved domains which may condition a high degree of cross-reactivity among botanically unrelated foods [3,4]. In addition to these proteins commonly involved in food allergy, profilins from pollens and plant foods as well as carbohydrate determinants (CCDs) can induce IgE cross-reactive responses in pollen-allergic patients [5,6].

In spite of the enormous advances in the knowledge of IgE food allergy the treatment is still based on the avoidance of the offending food. In fact there is not an effective treatment yet [7]. However, in cases of the most common food allergens avoidance is difficult to accomplish, and severe reactions from accidental exposures occur too often [8]. The first attempts of specific immunotherapy were performed with peanut extract by subcutaneous route, but with a high ratio of adverse reactions [9]. Oral and sublingual desensitization strategies were more recently tried for milk, egg, peanut and hazelnut allergies [8]. Despite the encouraging results, these therapies were attempted in patients allergic to a specific group of food allergens. However many patients suffering from food allergy are sensitised to panallergens which cross-react within an extensive group of different foods.

Polysensitization to a wide range of food allergens is common in some patients, particularly in those reacting to vegetables. Avoidance is extremely difficult and can induce severe dietary restrictions with consequent metabolic imbalance. These patients usually have a higher risk of increasing severity of symptoms and, potentially enhance the range of vegetable food sensitizations.

## Case presentation

During pregnancy a 36-year-old portuguese caucasian female patient started recurrent crisis of urticaria/angioedema, and three months after the delivery she underwent an allergological study. She referred a wide range of symptoms 15-20 minutes after ingestion of the majority of vegetable foods: oral allergy syndrome (OAS), urticaria/angioedemea, skin flush or rhinoconjuntivitis requiring high doses of systemic H1 antihistamines, so a severe dietary restriction was established. The allergological evaluation including commercial skin prick tests (SPT, Bial/Aristegui-Spain), prick-prick with fresh foods, and serum specific IgE (Phadia-Sweden) demonstrated a polysensitization to almost all vegetables food (fresh fruits, nuts, legumes, cereals, spices in others). Interestingly, the intensity of SPT reactivity and the serum specific IgE levels showed changes along the time of each 6 months clinical evaluation. They increased or decreased according to the specific food intake. The strictly restriction of a group of foods resulted on a decrease of sensitization to them however a progressive increasing sensitization was observed to those other vegetables foods better tolerated on the moment of setting up the diet and as a consequences not withdrawn from it. This oscillating clinical profile during a period of 4 years was implicated on metabolic disorder (hypertension, hypoalbumin and hypercholesterol serum levels). At the age of 40 years old she was submitted to a study that results on a specific treatment.

### 1 Allergological study

Skin prick tests confirmed the sensitization to a wide range of vegetable foods and no sensitization to most common aeroallergens. Total IgE was 70 UI/mL, and serum specific IgE levels confirmed the SPT reactivity. Specific IgE against peach and peach LTP (rPru p 3) were 1.5 kU/L and 3.5 kU/L respectively whereas negative values (<0.35 kU/L) were obtained for peach profilin (rPru p 4), birch profilin (rBet v 2) and CCD (Phadia, Upsala, Sweden).

The double-blind placebo-controlled food challenge (DBPCFC) to peach, according previous description of Fernández-Rivas *et al*. [10], was positive at first dose with OAS and rhinitis, and promptly remitted under convenient parenteral treatment.

Regarding these clinical and laboratory data the patient underwent an individualized treatment using a specific allergen which we were strong convinced that was responsible for the etiopathogenesis of the allergic vegetal food disease.

### 2 Methodology

The study was performed with the approval of the hospital ethics committee, and the written consent of the patient. All the clinical evaluation and laboratory measurements were performed at the beginning of the treatment period, and at 4, 8 and 12 months after the beginning. The tolerance of the treatment was observed during the immunotherapy period and the integrity of the immune system were evaluated by analysis of blood cell counts, blood biochemistry, liver enzyme studies, ionogram, glucemia and renal function.

#### Peach LTP (nPru p 3) purificaction

Peach peel was homogenated into Björksten [11] buffer and after centrifugation the supernatant was dialysed against 20 mM phosphate, pH 6), filtered (0.22 mm) and applied onto a 5 mL Capto S cationic column using an Akta prime system. Bound proteins were eluted with 20 mM phosphate, pH 6; 1 M NaCl, at a flow rate of 5 mL/min. Further purification was achieved by gel filtration using a Superdex 75 16/60 column equilibrated with PBS buffer. nPru p 3 was obtained at the 10 kDa fraction.

#### SPT

The SPT were performed in duplicate on the volar surface of the forearm according to standard procedure [12] with native Pru p 3 at the following concentrations: 1, 10, 100 and 500 μg/ml, with prick lancettes (Stallergenes, Antony, France) using one sterile lancette for each test. Histamine phosphate (10 mg/ml) and sterile serum saline (Bial/Aristegui, Spain) were used as positive and negative controls, respectively. Wheal areas were marked 15 minutes after the puncture with a fine-tipped ball-point pen and transferred by transparent adhesive tape onto paper for subsequent evaluation.

#### Total and specific IgE, IgG, IgG_1_ and IgG_4_

Total IgE was determined by the CAP System IgE FEIA (Phadia, Sweden). Serum specific IgE to nPru p 3 was measured by means of EAST technique (Enzyme Allergo Sorbent Test) and IgG against the same allergen by ELISA method [13].

#### DBPCFC

As previous referred challenge foods (masked taste) were prepared immediately before the challenge, and 3 doses (one eighth, one fourth, and five eighths of total weight of 150 g of fresh peach (peel and pulp) were administered at 1-hour intervals. The test was conducted in the hospital setting, with careful medical monitoring of the patient, and full emergency treatment was readily available.

#### Specific sublingual immunotherapy (SLIT)

The active specific immunotherapy consisted of native Pru p 3 at only one concentration of 40 μg/ml in NaCl 0.09%, Phenol 0.5% and 50% glycerol. The patient was instructed to keep the allergen solution in the mouth for at least 3 minutes and then swallow. The build-up phase was performed in an ultra-rush schedule and was completed in one day; it was administered in a one drop step every 30 minutes from 1 drop (2 μg) to 5 drops (10 μg) giving a cumulative dose of 30 μg of nPru p 3 in the first day. A maintenance dose of 10 μg per day (5 drops) and administered 5 times a week was established, obtaining a cumulative dose of 200 μg of nPru p 3 per month.

The build-up phase and the diary sublingual administrations during the first week of treatment were performed in a hospital setting with the availability of complete resuscitation equipment and trained personnel, and the patient was kept under constant observation after each administration and for at least 240 minutes after the last one.

All the rest of the treatment was accomplished by the patient alone without any kind of special care on the treatment administration.

Evaluation period of SLIT was from October 2007 to October 2008, and she was yet under treatment. During maintenance phase patient attended clinical observation once every 15 days.

### 3 Results

SLIT with nPru p 3 was safe and well tolerated because it did not elicit any adverse reaction. The metabolic parameters namely cholesterol, albumin and triglycerides returned to normal values. During the build-up phase the first 3 doses induced oral pruritus and paresthesias of tongue and lips however, increasing the doses with no additional pharmacological treatment was decided. We also emphasize the excellent adaptation of the patient to treatment, and the full compliance with the schedule of allergen administration and clinical and laboratorial tests.

Regarding the allergologic study, a reduction of the mean diameter in the SPT to nPru p 3 was observed, whereas no relevant changes on serum specific IgE, IgG, IgG_1_ and IgG_4_ to nPru p 3 were detected (Table 1). Besides the excellent clinical and SPT responses a non-expected maintenance of nPru p 3 IgE and IgG levels were observed after twelve months of treatment.

**Table 1.** SPT results to various concentrations of nPru p 3, levels of serum specific IgE, IgG, IgG_1_ and IgG_4_ against nPru p 3, and DBPCFC before SLIT, and at the 4^th^, 8^th^ and 12^th^ month of active treatment.

	T = 0	T = 4 months	T = 8 months	T = 12 months
**SPT** (mm)				
Histamine	6	6	7	8
nPru p 3 1 µg/ml	3	3	2	2
nPru p 3 10 µg/ml	6	4	3	2
nPru p 3 100 µg/ml	12	5	4	3
nPru p 3 500 µg/ml	14	6	5	4
**sIg levels**				
sIgE Pru p 3 (kU/L)	1.8	1.7	1.2	1.9
sIgG Pru p 3*	0.76	0.94	0.84	0.76
sIgG1 Pru p 3**	0.60	0.74	0.71	0.62
sIgG4 Pru p 3**	0.83	1.01	1.04	0.75
**DBPCFC**	Positive	Negative	Negative	Negative

*Absorbance at 492 nm with 1:200 diluted serum

**Absorbance at 492 nm with 1:50 diluted serum

***Absorbance at 492 nm with 1:100 diluted serum

Regarding the clinical efficacy, DBPCFC was positive at the first dose (18.75 g) before SLIT, inducing SAO and rhinitis, and it was negative at a cumulative dose of 150 g (maximum dose, 93.75 g) since the first challenge tests within the treatment period (4 months). We stress that the clinical symptoms improved steadily after the first month of SLIT, and by the 3rd month she had no major vegetables dietary restrictions, except for nuts, fruits and pepper. The antihistamines consumption markedly reduced. She was advised to avoid any nuts and pepper. Nevertheless when accidental intake occurred she only reported slight SAO symptoms, the majority with no need for medication.

## Discussion

This case report describes a patient with clinical sensitization to a wide range of vegetables food with tremendous consequences on the diet and metabolic imbalance, and not efficiently controlled with high doses of antihistamines.

The severity of the allergic symptomatology increases since the first symptoms appeared so our goal was to develop a specific immunotherapy for this case. Specific immunotherapy to foods have been tried using protein extracts obtained from the culprit raw sources [7]. We decided to design an immunotherapy treatment with the purified cross reactive allergen present in all the allergenic vegetables food.

SPT reactivity to various concentrations of nPru p 3 was carried out to look for the suitable immunotherapy allergen concentration. 10 μg/ml of nPru p 3 was the allergen concentration needed to induce a mean wheal similar in size to the one induced by histamine at 10 μg/ml. Taking this results into account and the manufacturer previous experience, a concentration of 40 μg/ml of Pru p 3 was chosen as the immunotherapy concentration [14]. This is a high dose of SLIT as the monthly cumulative maintenance dose is 5 ml at 40 μg/m (200 μg), compared to the conventional subcutaneous immunotherapy (SIT) in which the monthly dose is 1 ml at 10 μg/ml (10 μg) [15]. So it implies a cumulative dose 20 times higher for SLIT than to SIT.

The use of an ultra-rush schedule probably reduced the risk of side effects and explains the excellent tolerance both in the build-up and maintenance phases. The minor local symptoms occurring on the first 3 doses were not so serious to require administration of relive medication or the interruption of the treatment.

This clinical case confirmed that this treatment is safe, at least at a concentration that we used, was well tolerated and extremely effective on clinical symptoms. A tolerance to the intake of the majority of vegetable food sources was reached, the DBPCFC were promptly negative, and an improvement of the metabolic imbalance was obtained. At the moment, only nuts and particularly pepper induce SAO symptoms. It is still unknown if pepper has large amounts of LTP, besides the ability to induce specific IgE mechanism [1]. The decreasing SPT results to Pru p 3 are in agreement with the clinical response. However the specific IgE and IgG levels did not show significant differences as expected. The patient is still under treatment and we believe that more pronounced immunological changes will be observed in future.

## Patient’s perspective

I real fell well with this treatment and my life really improved, because I have no limitations on my diet and my health markedly improved since doctors started this knew treatment for my allergic disease.

## List of abbreviations

CCD: carbohydrate determinants
DBPCFC: double-blind placebo-controlled food challenge
LTP: Lipid transfer proteins
SLIT: specific sublingual immunotherapy
SPT: skin prick test
